# Regional differences in mortality in Greece (1984–2004): The case of Thrace

**DOI:** 10.1186/1471-2458-8-297

**Published:** 2008-08-23

**Authors:** Panagiotis Papastergiou, George Rachiotis, Konstantina Polyzou, Christos Zilidis, Christos Hadjichristodoulou

**Affiliations:** 1 Department of Hygiene and Epidemiology, School of Medicine, University of Thessaly, Larissa, Greece

## Abstract

**Background:**

Mortality differences at national level can generate hypothesis on possible causal association that could be further investigated. The aim of the present study was to identify regions with high mortality rates in Greece.

**Methods:**

Age adjusted specific mortality rates by gender were calculated in each of the 10 regions of Greece during the period 1984–2004. Moreover standardized mortality rates (SMR) were also calculated by using population census data of years 1981, 1991, 2001. The mortality rates were examined in relation to GDP per capita, the ratio of hospital beds, and doctors per population for each region.

**Results:**

During the study period, the region of Thrace recorded the highest mortality rate at almost all age groups in both sexes among the ten Greek regions. Thrace had one of the lowest GDP per capita (11 123 Euro) and recorded low ratios of Physicians (284) per 100 000 inhabitants in comparison to the national ratios. Moreover the ratio of hospital beds per population was in Thrace very low (268/100 000) in comparison to the national ratio (470/100 000). Thrace is the Greek region with the highest percentage of Muslim population (33%). Multivariate analysis revealed that GDP and doctors/100000 inhabitants were associated with increased mortality in Thrace.

**Conclusion:**

Thrace is the region with the highest mortality rate in Greece. Further research is needed to assess the contribution of each possible risk factor to the increased mortality rate of Thrace which could have important public health implications.

## Background

Variations and differences in terms of mortality are widely recognized both at national and international level [1]. In particular, mortality inequalities across geographical localities are well documented in various developed countries [2]. These differences could be attributed to a variety of factors including genetic, lifestyle and environmental [3]. Better understanding of regional mortality variations is important because it sheds light on the how well the aims of public health are being achieved, in addition it could provides hypotheses for further testing with the goal of better understanding disease aetiology, and thus improving preventative efforts.

Previous studies conducted in Greece referred to geographical variations of maternal mortality, and variations of some types of cancer [4-7]. To our knowledge there is no published study related to comparative assessment of regional crude mortality rates, and their determinants. Therefore, the aim of the present study is to compare different regions of Greece in terms of crude mortality and also to explore possible risk factors like: the low gross domestic product (GDP) per capita, low ratio of physicians and dentists per 100 000 population, the ratio of hospital beds/100 000 population and the percentage of Muslim population.

## Methods

Crude death rates (CDR) were calculated by gender in each of the 10 regional areas in Greece. Data regarding deaths were obtained from the annual series of the vital statistics of Greece – Deaths of the population ("Natural movement of population") during the period 1984–2004 (21 years). The total population in each region was derived from the 1981, 1991, 2001 Census of the national statistical service of Greece.

For both gender we used five-year intervals for the population age and the outcome was 18 age-categories. For each age-category for both genders we calculated the average of the yearly standardized mortality rates (SMR) for each region for the under study 21 year period (1984–2004). We used the method of direct standardization with respect to population census of the years 1981, 1991, 2001.

Data regarding hospital beds, doctors per 100 000 inhabitants, GDP per capita per region, specific mortality rates, and vaccination coverage were obtained from national statistical service of Greece. Quantitative data were presented by using median and interquartile range (IQR).

### Statistical analysis

All data were entered in a specially designed a database, and statistical analysis was performed using SPSS 11.0 software. Mann Whitney test was used to compare mortality rates of Thrace to these of other Greek regions, while chi-square test was used to compare vaccination coverage between Thrace and other regions. Multivariate regression models were used in order to assess factors independently associated with mortality rate. In these models mortality rate was the dependent variable, while possible risk factors like GDP, hospital beds, and doctors/100000 population were the independent variables. The level of statistical significance was set at 0.05.

## Results

**Figure 1:**
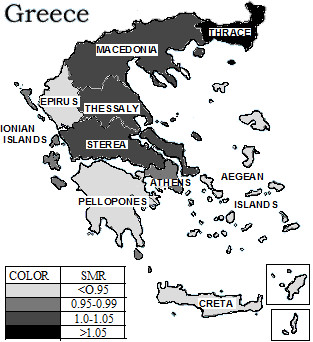
Regional distribution of mortality risk in males, Greece (1984–2004).

**Figure 2:**
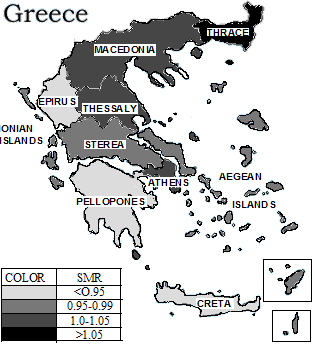
Regional distribution of mortality risk in females, Greece (1984–2004).

Among males and females at almost all age categories the region of Thrace recorded the highest age-specific crude mortality and standardised mortality rate (Figures 1, 2). The previously described trend of increased mortality of Thrace did not change during the twenty one years studied.

The most notable differences in terms of mortality rates between Thrace and national mean mortality rate were found at the age categories 0–4, 5–9, and 10–14 years.

### Age group 0–4 years

Among males at age category 0–4 years the regional mortality in Thrace was the highest in Greece with 345 deaths/100 000 inhabitants in comparison to the national mortality rate (mean) of 200/100 000 inhabitants. The region with the lowest mortality rate was Ionian islands (149/100 000) followed by Aegean islands (155/100 000) and Crete (161/100 000). Among females at that age group Thrace recorded the highest CDR in Greece (268/100 000) while the national CDR (mean) was 166/100 000. The region with the lowest CDR was Aegean islands (119/100 000) followed by Crete (127/100 000) and Sterea (133/100 000). At age group 0–1 Thrace recorded the highest CDR in comparison to the rest of Greece (median: 978, IQR: 828–1311) versus 651, IQR: 500–784, Mann-Whitney test, p < 0.001).

### Age group 5–9 years

The CDR in Thrace among males at the age category 5–9 years was well above the national CDR (33 deaths/100 000 vs 18/100 000, respectively). The lowest mortality rate was found in Ionian islands (13.1/100 000) followed by Athens (13. 5/100 000) and Epirus (15.8/100 000). Regarding females Thrace found again to have the highest CDR (23/100 000; national mean CDR: 14/100 000). Athens had the lowest regional mortality (9.8/100 000) followed by Aegean Islands (12.4/100 000).

### Age group 10–14 years

Mortality in Thrace among males was the highest regional in Greece with 36 deaths per 100 000 in comparison to national mortality rate (mean) of 20/100 000. Peloponnesus recorded the lowest regional mortality rate (11.5/100 000) followed by Athens (11.9/100 000). A similar pattern of regional mortality was revealed with respect to female population (CDR in Thrace: 26/100 000; national mean CDR: 14/100 000; Peloponnesus found to have the lowest CDR followed by Athens (11.5/100 000, and 11.9/100 000, respectively).

### Age groups 15–19, and 20–24 years

Among males at the age category 15–19 years Thessaly recorded the highest mortality rate followed by Ionian islands, and Thrace which recorded a CDR of 85 deaths/100 000 inhabitants (national mean CDR: 72/100 000). A similar pattern of regional mortality was emerged with respect to age category 20–24 years. In addition, it should be noted that Thrace recorded significantly higher mortality rates compared to the rest of the Greek regions in terms of some age groups.

This was the case (apart from 0–1 age group which has been already mentioned) for age group 1–4 years (median of Thrace: 62.95; IQR: 34.97–83.94 vs 26.28; IQR: 23.09–37.8 for the other regions (IQR: 18.54–42.36; p < 0.001), and 5–14 years (median of Thrace: 23.21; IQR: 17.02–33.77) vs 16.51 (IQR: 12.53–20.93; p = 0.018).

### Possible risk factors: GDP per capita, ratio of doctors, and hospital beds per 100 000 inhabitants

The lowest mortality rate both in male and female has been recorded in the regions of Peloponnesus, Epirus and the island of Crete, followed by the regions of Ionian and Aegean islands. The regions of greater Athens and Macedonia – the regions with the highest population count in Greece- and the regions of Thessaly and Sterea have not demonstrated differences with respect to the mortality rate in comparison to the national SMR (Figures 1, 2 and Table 1).

**Table 1:** Comparison of CDR (age categories: 0–14) with GDP, hospital beds and physicians for both sexes and in all regions (1984–2004).

**Regions**	CDR age < 1 per 100 000 population (median/IQR)	CDR ages 1–4 per 100 000 population (median/IQR)	CDR ages 5–14 per 100 000 population (median/IQR)	Mean GDP per capita in years 2000–2002*	mean hospital beds/100 000 population years 2001–2002	mean physicians per 100 000 population years 2001–2002
**Greece**	**651 **(500–784)	**26.28 **(23.09–37.80)	**16.5 **(15.02–20.06)	**15411**	**476**	**449**
Hepirus	570 (308–896)	36.98 (8.92–51.78)	17.5 (12.52–20.94)	11994	435	463
Thessaly	526 (384–676)	32.36 (25.17–47.15)	19.29 (15.99–21.63)	11754	410	332
**Thrace**	**978 **(828–1311)	**62.95 **(34.97–83.94)	**23.21 **(17.09–33.77)	**10913**	**279**	**328**
Ionian	454 (317–675)	37.37 (13.06–52.24)	17.64 (11.76–19.78)	16478	466	320
Creta	567 (391–632)	23.46 (19.11–34.40)	19.7 (11.76–20.93)	14657	508	461
Sterea**	528 (489–602)	31.95 (25.21–37.81)	14.44 (12.49–21.18)	----	167	239
Macedonian	648 (445–783)	29.57 (24.29–33.89)	17.65 (16.34–20.810	12512	496	427
Aegean	422 (383–601)	24.38 (14.63–39.9)	15.83 (12.63–22.29)	15470	393	290
Pelloponess	635 (259–823)	29.7 (22.87–38.11)	15.69 (11.62–21.58)	12051	311	348
Athens**	753 (575–983)	18.54 (14.73–25.28)	13.62 (11.10–15.10)	19876^+^	684	649

*Gross domestic product, in euro (€).

**in some regions data were not available

+Data for the region of Attica in which Athens is included

Thrace had the lowest gross domestic product (GDP) per capita in Greece recorded in the year 2001 (11 123 million Euros). Furthermore the region of Thrace recorded low ratio of doctors (284/100 000), per population in comparison to the national ratio of 422 doctors and 111 dentists, respectively. In 1999 the ratio of hospital beds per 100 000 population was very low (268/100 000) in Thrace, the second lowest in country in comparison to national ratio of 470 hospital beds/100 000 population (Table 1) [8].

### Specific mortality rates

Specific mortality rates were higher in Thrace in comparison to the national rate of Greece. In particular, the ratio of specific mortality in Thrace/specific national mortality of Greece was 1.6 for Tuberculosis; 1.9 for hypertension; 1.7 for psychiatric mortality; 1.4 for congenital malformation; 1.1 for accidents, and 1.5 for suicide.

### Vaccination coverage

Thrace recorded the lowest vaccination coverage in comparison to other Greek regions. In particular, Thrace recorded vaccination coverage for Hib 71.1% (95% CI: 64.6–76.8) vs 85.4% (95% CI: 84–86.7%) for Greece (chi-square test; p < 0.01).

Regarding vaccination coverage for the fourth dose of DTP vaccine, Thrace recorded a significantly lower vaccination coverage in comparison to the national coverage of Greece (94%; 95% CI: 90.2–96.3 vs 98.3%; 95% CI: 97.7–98.8; chi-square test; p < 0.01).

### Multivariate analysis

Multiple regression analysis has shown that GDP was significantly associated (inverse association) with CDR in Thrace at age group 5–14 (β-coefficient = -0.001; p = 0.02; r = 0.59).

At age group 0–14 analysis has revealed that ratio of doctors/100000 population were significantly associated (inverse association) with CDR (β-coefficient = -0.359; p = 0.03; r = 0.413).

## Discussion

Descriptive analysis has indicated that Thrace was the region with the highest mortality in Greece for both men and women at almost all age categories. Furthermore, Thrace demonstrated mortality rates well above the EU average. There is a question about the aetiology of this increased mortality risk in Thrace. Our results indicate some possible risk factors: Thrace recorded the lowest regional GDP in Greece, and limited access to healthcare services. Multivariate analysis (age group 5–14) documented that GDP was a significant determinant of mortality rate in Thrace, in addition a significant association regarding ratio of doctors/100000 population was detected at age group 0–14. These notable findings could be taken into account in future planning in health care strategy, and in allocation of resources. In particular, the finding that the mean GDP of Thrace is very low compared to other Greek regions could have important implications in terms of policy making. However, the low GDP, and the low hospital bed per population ratio in Thrace deserve further attention and research.

In addition, the findings related to vaccination coverage, and specific disease rate could assist in understanding/explaining the regional mortality differences noted. In particular, the lower vaccination coverage observed in Thrace could be associated with increased mortality due to meningitis, and epiglottiditis.

However, other risk factors should be explored (genetic, lifestyle, occupational and environmental exposures) [9,10]. In addition, the high percentage of a Muslim minority in Thrace could represent a possible factor associated with increased mortality risk, but this hypothesis is not being supported by several studies which reported that at least for some types of cancer (breast, endometrial, ovarian) Muslims recorded significantly lower mortality rate in comparison to Christians Orthodox [5-7]. In addition, a study conducted in the prefecture of Xanthi (Thrace) revealed that Muslims present lower incidence rate of ischemic stroke than Christians. Also the outcome of hospitalization due to stroke did not differ significantly by religion [11].

It should be stressed that Thrace was partly except from the population exchange following the end of the Greek-Turkish war in 1922. Furthermore, there has been a significant amount of migration within Greece during the recent decades. It is possible that some of this will have been selective with healthier folk moving to healthier areas. However, if net migration patterns play a role regarding increased mortality in this region has to be confirmed by further research.

There appears to be a south to north trend in increasing mortality risk, but this trend was not reflected in the putative risk factor data.

Our study has some limitations by being descriptive, thus we can not provide information on causal associations between mortality and several possible risk factors. An additional limitation was the absence of regional data (e.g. on smoking habit, and diet). However, we performed multivariate statistical analysis, and – in part-some speculations have been statistically tested.

## Conclusion

Thrace presented the highest mortality risk among 10 regions in Greece, and in particular at age groups < 1 year; 1–4, and 5–14 years.

Further statistical analysis revealed that the low GDP and low ratio of doctors per population were significantly associated with the increase mortality in this region which could have important public health implications. The findings observed deserve further attention and research given that Thrace has a special geographic characteristic: it represents a "bridge-link" between two continents since it stands between Europe and Asia.

## Competing interests

The authors declare that they have no competing interests.

## Authors' contributions

PP participated to study design, data collection, statistical analysis, and manuscript preparation. GR participated to statistical analysis, preparation, and revision of the manuscript. KP participated to data collection, and preparation of the manuscript. CZ participated to study design, statistical analysis, and preparation of the manuscript. CH participated to and supervised study design, collection of data, statistical analysis, preparation, and revision of the manuscript. All authors read and approved the final form of the manuscript.

## Pre-publication history

The pre-publication history for this paper can be accessed here:


